# Four-dimensional joint visualization of electrode degradation and liquid water distribution inside operating polymer electrolyte fuel cells

**DOI:** 10.1038/s41598-018-38464-9

**Published:** 2019-02-12

**Authors:** Robin T. White, Sebastian H. Eberhardt, Yadvinder Singh, Tylynn Haddow, Monica Dutta, Francesco P. Orfino, Erik Kjeang

**Affiliations:** 1 Fuel Cell Research Laboratory (FCReL), School of Mechatronic Systems Engineering, Simon Fraser University, 250-13450 102 Avenue, Surrey, BC V3T 0A3 Canada; 2 Ballard Power Systems, 9000 Glenlyon Parkway, Burnaby, BC V5J 5J8 Canada

## Abstract

Understanding of degradation mechanisms present in polymer electrolyte fuel cells (PEFCs) is important to continue the integration of this clean energy technology into everyday life. Further comprehension of the interaction between various components during fuel cell operation is also critical in this context. In this work, a four-dimensional *operando* X-ray computed tomography method is developed for combined visualization of all PEFC components as well as transient water distribution residing in the cell, which results as a by-product of the electrochemical reaction. Time resolved, identical-location visualization through degradation stages is uniquely enabled by the non-invasive and non-destructive qualities of this method. By applying an accelerated stress test that targets cathode catalyst layer (CCL) corrosion, novel observations resulting from morphological changes of the CCL such as reduction in the water volume in the adjacent gas diffusion layer, CCL crack formation and propagation, membrane swelling, as well as quantification of local carbon loss is achieved. Additionally, insight into features that contribute to reduced fuel cell performance is enabled by the use of this specialized imaging technique, such as increased membrane undulation causing delamination and separation of the CCL from the microporous layer, which greatly affects liquid water pathways and overall device performance.

## Introduction

In recent years there has been considerable increase in the commercial adoption of polymer electrolyte fuel cells for power generation from hydrogen. This is owing to the high efficiency and low environmental impact of their operation; making them attractive as alternative power sources for automotive applications, material handling, stationary combined heat and power (CHP) applications, unmanned aviation vehicles and wherever high energy demands exist and short recharge intervals are desirable^1–6^.

**Figure 1:**
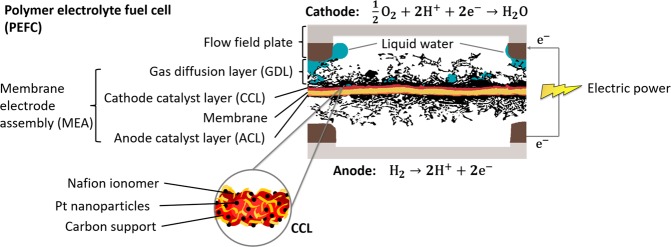
Schematic of polymer electrolyte fuel cell (PEFC) components obtained from XCT visualization of an operating fuel cell, outlining the electrochemical reactions, electronic transport and liquid water generation. The inset highlights a simplified illustration of the cathode catalyst layer composition and structure.

Polymer electrolyte fuel cells (PEFCs) are designed as a series of layers as shown in Fig. 1; beginning with a proton conductive and electron insulative polymeric electrolyte membrane at the center, which separates the anode and cathode electrodes that are coated on its opposite sides in the form of a nano-porous catalyst layer (CL) composed of platinum nanoparticles on carbon support intermixed with ionomer. The complete membrane electrode assembly (MEA) further comprises of two macro-porous gas diffusion layers (GDLs), each coated with a micro-porous layer (MPL) on the surface adjacent to the CL interface. Water is formed in the CCL as a by-product of the electrochemical cell reaction involving hydrogen oxidation at the anode and oxygen reduction at the cathode. While adequate hydration of the ionomeric membrane and CLs is desirable for maintaining high proton conductivity and low ohmic losses, excessive hydration also causes liquid water accumulation (or *flooding*) in the porous structures and limits the diffusion of reactants to the active sites in the CCL. Accordingly, effective water management across the multi-scale porous structures of the GDLs, MPLs and CLs, is vital for high fuel cell performance and durability^7,8^.

While considerable research efforts have been made to understand the liquid water distribution in the GDL in order to improve fuel cell performance^9–14^, investigations that encompass CCL degradation and its consequential changes to the GDL water distribution within an operating fuel cell are largely absent. During operation, the CCL can often undergo substantial structural, mechanical and chemical degradation associated with high cathode potentials and concomitant corrosion of all carbonaceous components, most importantly the catalyst support material^15–18^. Damage features as a result of this degradation are: crack formation, thickness loss, decrease in electrochemically active platinum surface area (EPSA) and changes in diffusive and electric pathways due to partial collapse of the porous carbon structure leading to increase in transport losses and decreased performance^19–22^. Additionally, generation of hydrophilic oxygenated carbon surface groups can cause increased flooding at the surface of the CL leading to further mass transport losses^16^. Accurate characterization of these damage features is important in order to relate any changes in liquid water distribution to electrode degradation, as well as properly understand the performance losses observed. Previously, Fairweather *et al*.^23^ used neutron imaging to investigate the impact of CL degradation on the water distribution inside an operating PEFC. Limitations associated with neutron imaging such as low resolution (13 µm spatial) and low material contrast, however, restricts the ability to clearly visualize and thus accurately correlate the structural changes within the CCL that may have impacted the water distribution and performance.

Lab-based X-ray computed tomography (XCT) has been shown to allow for high resolution (<3 µm) three-dimensional (3D) imaging of fuel cells^24–29^, while periodically performing same location tracking across degradation stages during a typical accelerated stress test (AST), thus adding a fourth imaging dimension (*i.e*., degradation time)^19^. This enables the quantification of various temporal changes in the electrode morphology during its degradation. Previously, only synchrotron based X-ray tomography has been used to investigate 3D liquid water distribution in the GDL of operating PEFCs, owing to the short scan time and high signal-to-noise ratio requirements^9,14,30–33^. Degradation of a fuel cell cathode is a slow process, even when using AST cycles with high (>1.2 V) upper potential limits, thus making the use of a synchrotron impractical with much of the allotted beam time devoted to running degradation protocols rather than image acquisition. However, the convenience of lab-based XCT allows for repeated image acquisition and visualization at studying steady state conditions, such as steady state water distribution at constant current density. The attenuation of X-rays is dependent on the mass attenuation coefficient (*µ*) and density (*ρ*) of the material^34,35^. In a material comprising of several phases, such as the CCL, this dual dependence of X-ray attenuation results in variation of greyscale values of pixels after reconstruction. Additionally, any relative changes in material composition or density can theoretically be observed in a degrading CCL by comparing greyscale changes, thus allowing for an opportunity to infer its material property changes. This dependence of X-rays on material composition has been suitably leveraged in scanning transmission X-ray microscopy (STXM) to obtain spatial distributions of carbon and ionomer within the catalyst layers^36,37^. However, SXTM suffers from specific limitations in terms of sample size requirements and housing, thus not allowing for *operando* imaging wherein the imaging is performed in a current-producing operating fuel cell.

**Figure 2:**
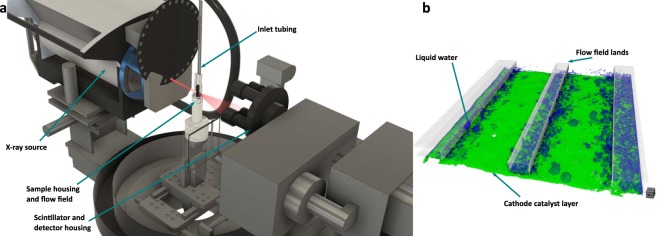
(**a**) Schematic of Zeiss Xradia Versa XCT system showing the customized small-scale fuel cell fixture and sample housing developed for operando imaging. (**b**) 3D segmented views highlighting the liquid water visualization and cathode catalyst segmentation. (For interpretation of the references to color in this figure legend, the reader is referred to the web version of this article).

Given their sufficient spatial resolution for characterizing CCL morphology, ability to periodically image under steady state fuel cell conditions, and accommodation of housing accessories needed for a fully operational fuel cell, lab-based XCT systems are presently the most suited instruments for simultaneously investigating CCL degradation and liquid water distribution along with their interaction. A small-scale fuel cell fixture, which allows for imaging the active area within a small field of view without strongly attenuating the X-ray beam in a lab-based XCT system, was designed and presented previously^19^. This experimental fixture is leveraged in the present work to perform four-dimensional *operando* visualization of fuel cell cathode degradation, see Fig. 2. To the best of the authors’ knowledge, the combined visualization of water distribution and cathode morphology is *first* published here. Additionally, post processing analysis to obtain compositional information on CCL changes following degradation is also presented here. Using these novel analysis methods, we present results of water distribution changes following CCL degradation, as well as compositional and morphological changes within the CCL itself. These results are further compared to fuel cell performance losses and supplementary diagnostic data that uncovers key pieces of information that may aid in the development of next generation fuel cell electrodes.

## Results

### Water distribution changes following catalyst layer degradation

**Figure 3:**
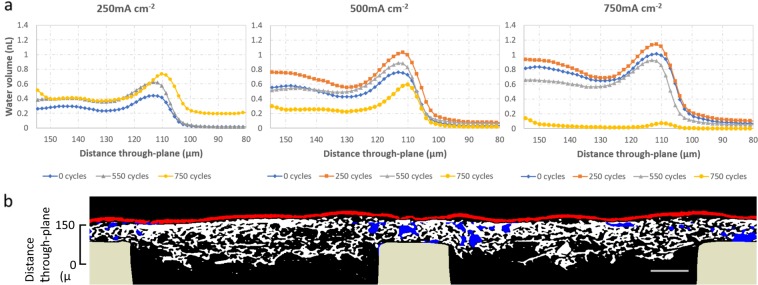
4D operando liquid water visualization and assessment by XCT. (**a**) Quantified distribution of liquid water volume through the cathode GDL thickness at various current densities and at different degradation stages of the voltage cycling AST. (**b**) Segmented cross-sectional visualization of liquid water (blue) in the GDL (white), cathode catalyst layer (red), and lands (beige). The scale on the left identifies the location of water quantification in (**a**). (For interpretation of the references to color in this figure legend, the reader is referred to the web version of this article).

Combined visualization of transient water distribution and CCL degradation by 4D *operando* imaging is presented for the first time. At the beginning of life stage, near the surface of the flow-field lands, under the conditions chosen, considerable volumes of liquid water are pooled by the reduced gas flow and decreased porosity by compressed GDL pores. Some droplets also reside on the surface of the GDL and are fed by connected flow networks from the CL surface. These droplets are stable enough to maintain their volume during the course of the imaging period. Following degradation, a significant change in the water content of the GDL is observed, as a result of significant carbon corrosion from voltage cycle AST. The location of most of the water pooling was situated under the lands of the flow field, which remained consistent throughout cycling and current density measurements, however the amount of water changed steadily. Shown in Table 1 is the saturation calculated under land and channel at 0 cycles (beginning of life) and at 750 cycles representing a heavily degraded state. This value was calculated by averaging the ratio of water volume to total pore volume for each 1.5 µm step of the 3D image stack. At 0 cycles, the obtained value of 0.37 is in good agreement with other saturation studies^38^. The porosity used for this calculation of the GDL under land and channel is shown in Supplementary Information (SF1). At 0 cycles, the water content in the GDL follows an expected trend, with steadily increasing water volume with increasing current density^10^, as shown in Fig. 3. The pattern for water pooling demonstrates high amounts of liquid droplets on the surface of the GDL and near the surface of the land (approximately 120 µm on the x-axis), followed by a decrease moving through the GDL, and then another slight increase following the change in GDL structure as porosity decreases from invasion of the MPL. Upon initial cycling by AST and again measured at 250 cycles, the trend is consistent with beginning of life (BOL), however, with higher relative water volume at 500 mA cm^−2^ and 750 mA cm^−2^. Following an additional 300 cycles, there is a turning point to a change in behavior of the water content in the MEA. At 250 mA cm^−2^ the water content is comparable to BOL, however for the higher current densities of 500 and 750 mA cm^−2^ there is a noticeable decrease in the water volume compared to the previous cycling stage. This trend continues with further cycling up to 750 cycles, making the change highly apparent, with almost all liquid water being removed for 750 mA cm^−2^ at a highly degraded state. This trend is consistent with previously reported results using neutron imaging by Fairweather *et al*.^23^ which used the same voltage cycling procedure. Fairweather *et al*. imaged at constant voltage of 0.6 V, which corresponds approximately to 750 mA cm^−2^ operation for this study at high degradation stage. The addition of lower current density measurements presented here and the favorable resolution of the XCT technique provides further insight into the change in fuel cell behavior, as well as additional detailed imaging of the CCL changes.

**Table 1:** Saturation calculation in the cathode GDL region under the lands and channels of an operating PEFC at 750 mA cm^−2^ at the beginning-of-life (0 cycles) and end-of-life (750 cycles) degradation stages.

Degradation stage	Saturation - Land	Saturation - Channel
0 cycles	0.37 ± 0.04	0.01 ± 0.01
750 cycles	0.009 ± 0.003	0.001 ± 0.003

### Catalyst coated membrane movement following cathode degradation

**Figure 4:**
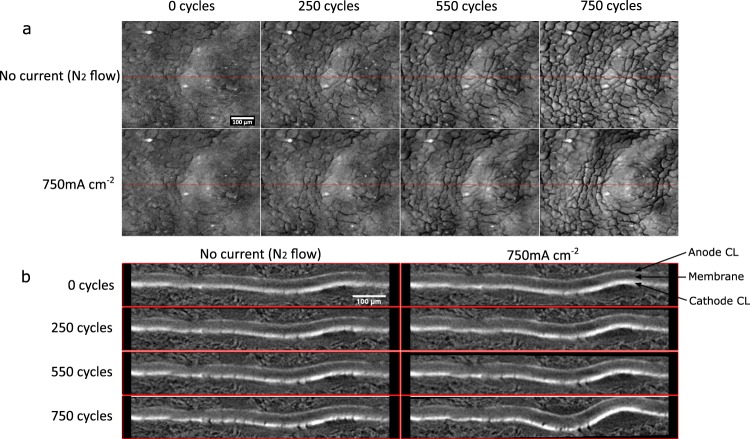
4D operando images featuring (**a**) planar and (**b**) cross-sectional virtual slice views of the CCL and MEA, respectively, during ‘dry’ state (No current) and ‘wet’ state (750 mA cm^−2^) at various degradation stages (as indicated by the cycle numbers), highlighting CCL crack formation and propagation and increased stress-induced membrane movement resulting from its expansion under hydrated conditions.

A unique observation afforded by the presently adopted visualization methodology, which involves XCT-based 4D *operando* imaging, is the identification of hydration-induced movements of CLs and membrane following stress cycles. As shown in Fig. 4a (top row at no current), CCL crack formation and propagation are observed due to the corrosion of carbon support in the catalyst layer as well as from possible movement of the membrane. These images are acquired at room temperature with only nitrogen gas flow (100% RH at 25 °C) and without any hydration-inducing current withdrawal from the cell; accordingly this state of imaging is termed as a ‘dry’ state. Once the cell is operated with hydrogen and air under an applied load, water is produced leading to membrane water uptake and expansion^39^. As a result of this expansion, mechanical stresses are generated within the constrained membrane, forcing undulations due to buckling^34,40^. As can be seen in Fig. 4a (bottom row at 750 mA cm^−2^), the CCL cracks can locally open or close depending on the bending direction within the adjacent membrane. The amount of bending in the through-plane direction is found to be substantial, particularly at highly degraded states, when observed using the cross-sectional views shown in Fig. 4b. It is interesting to note that the amount of membrane movement/bending in the ‘wet’ state increases following the degradation cycles, however, the ‘dry’ state positions are largely unaltered from BOL to 750 cycles, with the CLs and membrane returning to approximately the same position. These membrane undulations can produce local regions of separation between the CCL and MPL, thereby creating void spaces that may compromise the cell’s water management functionality and promote local flooding. Moreover, the force applied from the through-plane movement of the membrane may produce locally over-compressed regions within the GDL, which may impact the porosity and consequently their liquid/gas transport properties.

### Carbon support degradation

**Figure 5:**
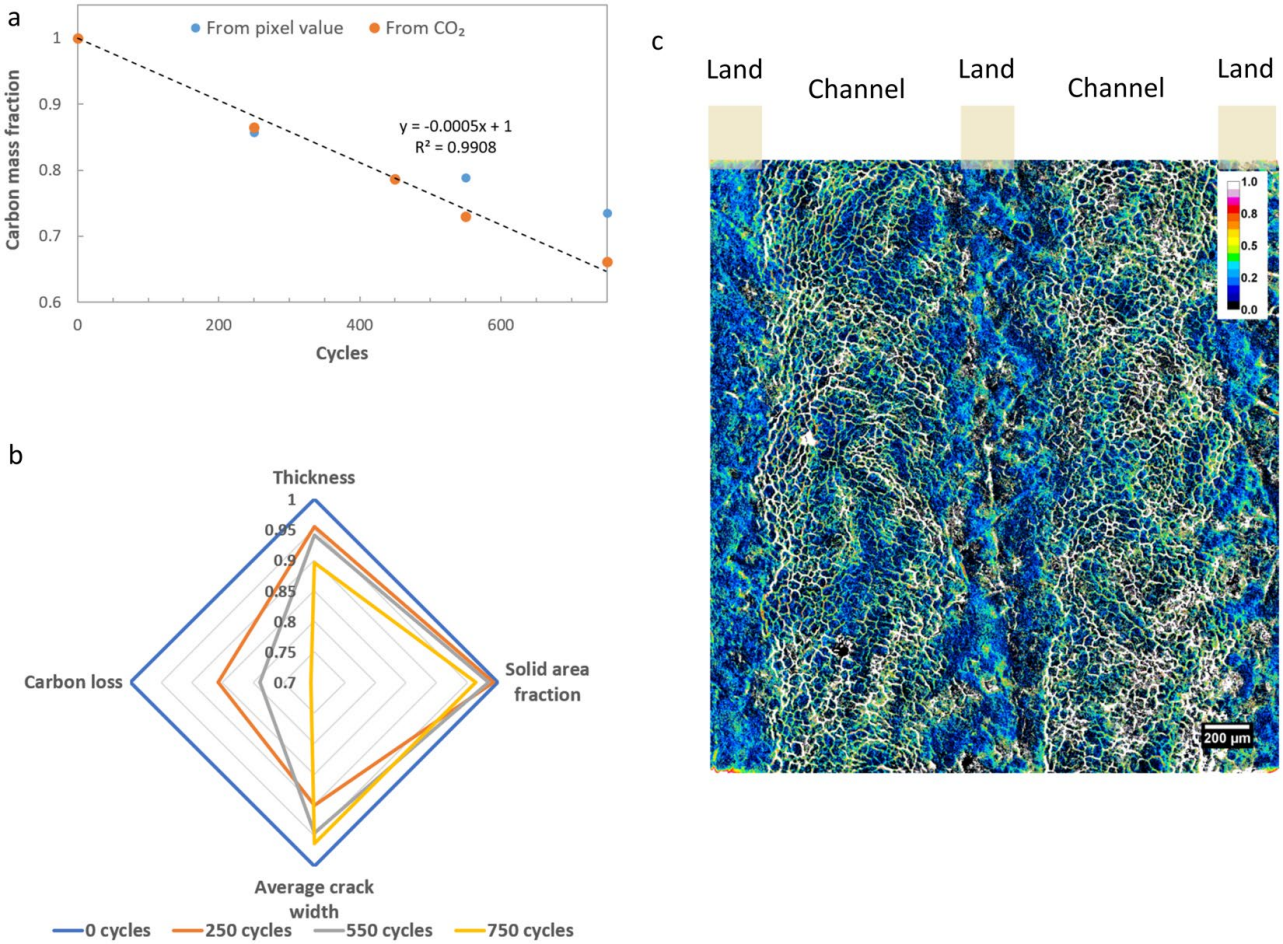
Quantification of morphological feature changes in the CCL through calculation of carbon loss from the changes in pixel greyscale value of the XCT datasets. (**a**) Comparison of calculated carbon loss with experimentally measured CO_2_ output. (**b**) Comparison of morphological changes in the CCL during degradation with the changes in carbon loss. (**c**) Spatial distribution of fractional local carbon loss in the CCL from BOL to 750 cycles, shown through a pseudo-color overlay on a planar/top-down view of the CCL imaged by XCT. (For interpretation of the references to color in this figure legend, the reader is referred to the web version of this article).

Due to the applied cathode potentials above 1.0 V_RHE_, carbon support material in the CCL undergoes oxidation and is lost in the form of CO_2_/CO. This carbon corrosion weakens the catalyst structure and can eventually lead to crack formation and collapse in thickness^8,16,19,41^. After segmentation of the CCL from the measured XCT data, quantification of morphology changes following degradation of carbon support can be obtained, such as CCL thickness, crack area and crack width. In addition, a local measure of the carbon loss can be calculated by using relative changes in the pixel greyscale value. Previously, it has been shown that attenuation changes arise from degradation of the CCL, suggesting that a calculated CCL composition could be obtained along with its spatial and/or temporal distribution^19^. These results are highlighted in Fig. 5a, where the calculated carbon loss (model) is validated against carbon loss measured from CO_2_ output in the exhaust gas. The carbon loss calculation from the XCT data is found to be in good overall agreement with the CO_2_ measurements. Some deviation from experimental values occur toward higher cycle numbers and may indicate inaccuracy resulting from the optimization search, as the calculated values have a larger deviation from nominal BOL values. It can also suggest however, that carbon loss from other sources besides the CL may be contributing toward the measured CO_2_^42^. Shown in Fig. 5b is the comparison of morphological values with the obtained carbon loss. It is noted that a higher proportion of carbon is lost, altering the internal structure, than can be accounted for simply by reduction in thickness or crack formation. The relative local carbon loss is shown in Fig. 5c, where the highest fraction is observed to correspond to crack formation where all solid is lost in this area, most notably occurring under the channel regions.

**Figure 6:**
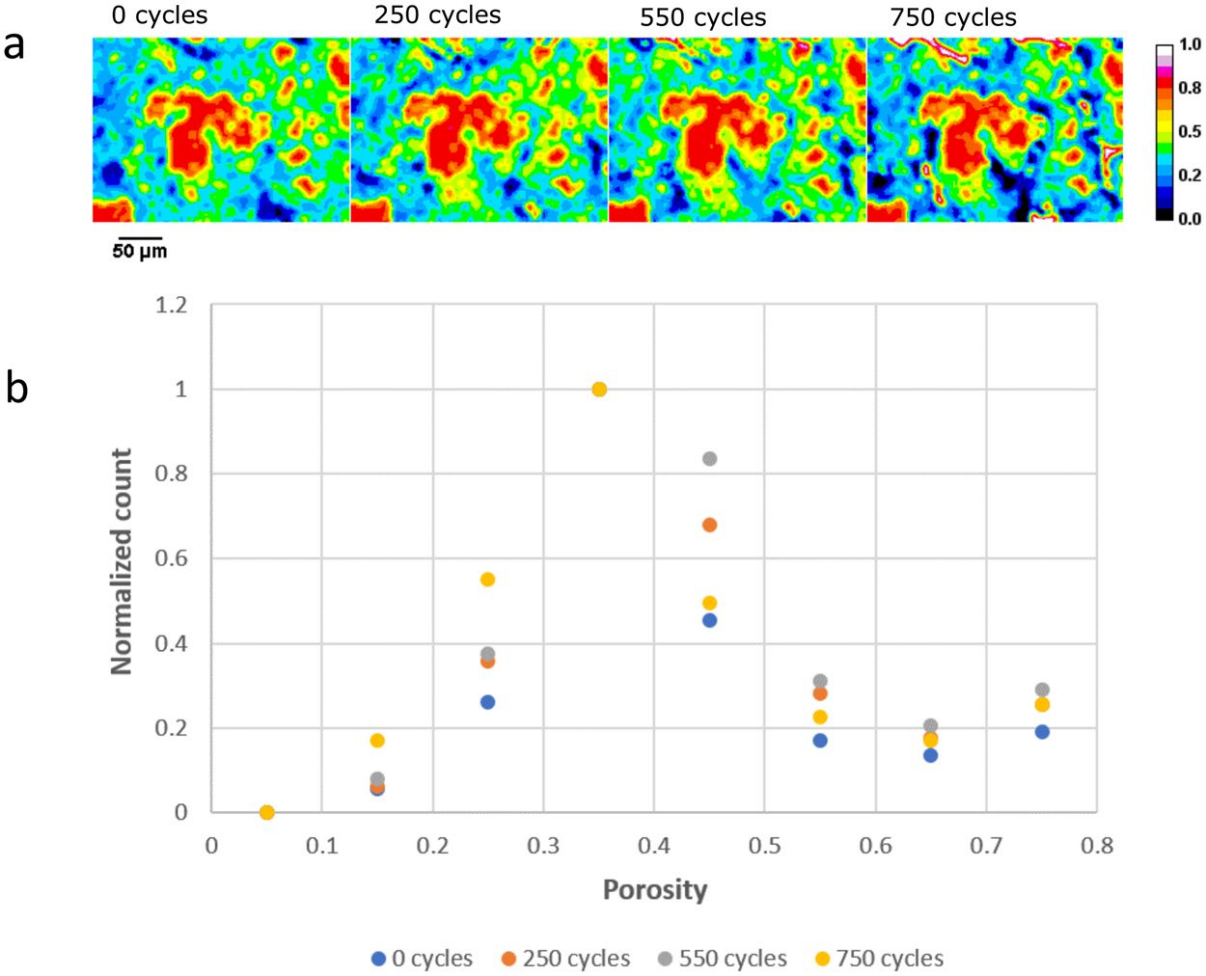
Quantification of porosity changes in the CCL during the voltage cycling AST, obtained through calculations based on pixel greyscale values. (**a**) Spatial distribution of porosity within the CCL plane shown with pseudo-color from a subset of the full-FOV. (**b**) Histogram plot showing the distribution of porosity values across the entire CCL FOV. (For interpretation of the references to color in this figure legend, the reader is referred to the web version of this article).

From the calculated local carbon loss, other properties such as local porosity can also be obtained, with the spatial porosity distribution and overall histogram shown in Fig. 6a,b, respectively, as a function of degradation. As seen from the images and histogram, there is an incremental shift toward higher porosity until advanced stages of degradation, where the shift is reversed back toward low porosity, even below the original value at BOL. These results suggest a balance between CCL thickness and internal structure changes where internal corrosion can lead to increase in porosity (decrease in density) until the reduction in thickness is sufficient to cause collapse and reduce porosity (increase density)^17^. From local thickness measurement and composition distribution calculation, the perceived collapse is local at this stage of degradation with regions of variable ionomer content and thickness behaving differently than other regions. Following even further degradation, more uniform thickness will be present as a larger fraction of the catalyst area undergoes this collapse. This locally variable degradation stage will also play a role in heat generation, water production, and gas flow; all affecting the impact of local performance of the catalyst layer.

### Fuel cell performance

**Figure 7:**
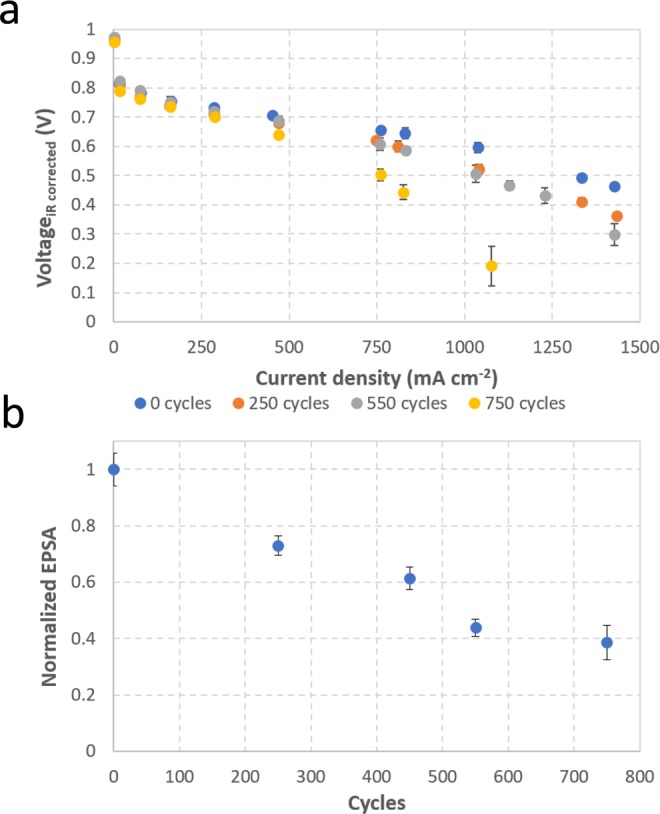
(**a**) Polarization curves and (**b**) cathode electrochemically active platinum surface area (EPSA) for the small-scale fuel cell at various degradation stages of the voltage cycling AST. The EPSA data shown are normalized against the BOL value.

In addition to the combined visualization of CCL degradation and liquid water distribution, fuel cell diagnostic information was also collected to monitor changes in electrochemical performance. Figure 7 shows the performance at various degradation stages as well as changes in the EPSA at the cathode from the small-scale fixture used for imaging. From the observed results, following stages of degradation by AST, a significant increase in mass transport loss occurs after 750 cycles. A more gradual and consistent decrease in EPSA occurs however, which follows good agreement with previous studies and relates well to the decrease in carbon support by corrosion and other damage features such as cracking^16,19,43^.

## Discussion

The combined visualization of water distribution and CCL degradation in a fuel cell, enabled by a customized XCT-based 4D *operando* imaging methodology and advanced post-processing techniques, is presented. This concurrent analysis offers novel insight into the interactions between various components that comprise a fuel cell system. Fairweather *et al*. had previously suggested that the observed decrease in liquid water content in the GDL following cathode corrosion likely results from temperature increase caused by elevated levels of heat generation that accompany performance losses associated with degradation of the CCL^23^. Observations made during the present work, at a given current density, are consistent with this report. Additional observations such as CCL thinning and carbon loss also suggest localized increase in heat production that could more readily facilitate vapor phase water transport from the CCL. However, the increased membrane swelling and associated stress-induced movement observed at advanced degradation stages further suggests that the rate of water removal from the catalyst layer may still be relatively slow, leading to an increase in local humidification within the MEA and flooding within the CCL. Degradation-induced damage features, such as increased crack formation and MPL-catalyst layer separation in some locations caused by significant membrane movement, can generate void spaces that may promote liquid water pooling and induce mass transport losses by blocking the diffusive pathways for incoming oxygen. The onset of significant performance losses in the mass transport region, particularly at 750 cycles, correlate well with the observed visualization results, as well as the aforementioned hypotheses of water evaporation within the GDL and local flooding within the catalyst layer. In addition, a decrease in contact area from membrane undulation can further increase associated losses. An approximate area of MPL separation is measured between 15–20% at high degradation state with large membrane movement. The gradual decrease in EPSA with degradation is likely a cumulative result of: (i) loss in solid catalyst layer area caused by crack formations; (ii) reduced interfacial contact caused by carbon support corrosion; and (iii) local flooding inhibiting the transport of gases to active sites in the catalyst layer^7,43^.

The present calculations of porosity (from greyscale pixel values) and carbon loss (Fig. 6) correlate well with previous results from Star *et al*.^17^, wherein carbon loss percentage was compared to porosity changes in PEFCs using a voltage cycling AST similar to that employed in the present work. The calculated results also indicate progressive changes in catalyst layer structure resulting from corrosion stages, where the porosity value increases initially due to pore formation from the removal of carbon support until the reverse effect from collapse of support structure becomes more dominant leading to a decrease in the local porosity value. This decrease in porosity will impact diffusive properties of the catalyst layer and inhibit gas diffusion leading to an increase in mass transport losses. The decreased porosity can also impact the CCL’s water removal characteristics leading to additional flooding and increasing local humidity. Consequently, the ionomer membrane’s local environment is altered by these elevated humidity levels, which allows for higher membrane water uptake leading to the increased expansion, mechanical stresses and buckling, as shown in Fig. 4b. Table 2 shows the measured membrane thickness under both ‘dry’ N_2_ conditions without current and ‘wet’ conditions during operation at 750 mA cm^−2^. The thickness at ‘dry’ conditions shows no observable change from BOL up to 750 cycles, whereas the thickness under ‘wet’ conditions appears to increase gradually with the progression of CCL degradation. In particular, the ‘wet’ membrane thickness is highest at 750 cycles which coincides with the increased amount of membrane undulation observed at this stage. Goulet *et al*.^44^ had previously shown that the water uptake capacity and hydration-induced thickness change of the membrane does not vary much between the cases of: (i) presence and (ii) absence of bonded electrodes. Therefore, any degradation-induced variation in the restraining of membrane by the bonded CCL that may lead to the observed thickness increase is highly unlikely. Instead, the hypothesis of enhanced local hydration of the membrane environment caused by catalyst layer flooding seems more feasible and agrees with the various observations made in this work.

**Table 2:** Membrane thickness measured from the operando XCT images at various degradation stages under both ‘dry’ and ‘wet’ states.

	Membrane thickness	
	at 0 current with N_2_ flow (µm)	at 750 mA cm^−2^ (µm)
0 cycles	20.8 ± 1.7	21.1 ± 1.8
250 cycles	20.5 ± 1.7	23.4 ± 2.0
550 cycles	21.5 ± 1.6	23.2 ± 2.3
750 cycles	20.9 ± 1.5	26.1 ± 2.7

The increase in membrane thickness under ‘wet’ states correlate well with the observed increase in membrane and CL movement resulting from hydration-induced mechanical stresses. Plus-minus values indicate variation in the thickness across the full FOV.

Cathode catalyst layer cracks were almost non-existent at beginning of life post-conditioning (see Fig. 4a). While the initial new crack formations reduced the average crack width from BOL to 250 cycles, as shown in Fig. 5b, further crack propagation and carbon corrosion brought back the average crack width closer to the BOL value. As observed in Fig. 4, significant movement results from expansion of the membrane at high current densities. In addition to the voltage cycling during the applied AST, it is plausible that the sequential ‘dry’ and ‘wet’ visualization scheme may also induce mechanical crack development due to the associated membrane movement. Given that the membrane movement from ‘dry’ to ‘wet’ states is negligible at the initial degradation stages and that the new crack formations seem to initiate during this period, the contribution of mechanical stresses/movement to the CCL crack development is likely to be marginal or negligible. This can be seen in the comparison of ‘wet’ state images of the cathode plane between BOL and 250 cycles (Fig. 4a), wherein the new crack formations observed at 250 cycles are evidently a result of the intermediate voltage cycling. Nevertheless, increased water retention in the catalyst layer with progressive degradation may give rise to such mechanical crack formations at the later stages of voltage cycling AST, when the membrane movement is more pronounced. It is further possible that hydrophilic species formation from the oxidation of carbon support could promote initial water retention, which could then trigger a chain-reaction like effect wherein higher water retention leads to both higher corrosion and greater membrane movement, collectively leading to increased rates of crack formation/growth and flooding.

## Conclusion

For the first time, combined visualization of CL degradation stages and water distribution in PEFCs is presented. Upon applying an AST to simulate possible cathode potential swings during PEFC operation, degradation of the CL from corrosion of carbon support is observed. In addition, visualization and quantification of water distribution in the GDL is obtained and is shown to change significantly over the lifetime when measured *in operando* at constant current. Surprisingly, at the end-of-life stage, liquid water in the GDL is no longer present at moderate to high current densities, which indicates evaporation due to heat generation. In addition, catalyst layer movement from stresses applied by membrane swelling by water absorption is visualized and shown to increase significantly at advanced stages of cathode degradation. This occurs as a result of catalyst layer flooding as a result of porosity change and additional hydrophilic surface groups introduced from corrosion of carbon support, in addition to crack formation allowing for areas with which liquid water can accumulate. Additional degradation features such as separation of the CL from the MPL as well as CL crack opening and closing is observed. These degradation features will inhibit proper water removal from near the CL leading to additional performance losses. Quantification of CL composition change by calculation from pixel greyscale values obtained by XCT is also presented and shows excellent agreement with carbon loss from carbon corrosion as measured by CO_2_ exhaust gas monitoring. In addition, this calculation allows for local carbon loss to be observed, as well as material property values such as porosity change to be calculated. During degradation, the CL porosity is first shown to increase before a sudden decrease at end-of-life. This signifies corrosion of the carbon support first leading to a hollowing effect eventually causing collapse of pores and increased density during the observed thinning of the CL at advanced degradation stages. This reduced porosity will also impact the removal of water and efficient operation of the CL with significant mass transport losses becoming apparent. From the combined visualization of water distribution in the GDL as well as *operando* visualization of the CL features and expansion behavior of the membrane, a strong interplay between MEA components is presented in operating fuel cells. With technological improvements in visualization methods and processing, significant advances into the understanding of degradation induced changes that result during fuel cell lifetime has been achieved.

## Methods

### *Operando* imaging and accelerated stress testing

The design of the small-scale fuel cell hardware used in this study has been previously presented, with details found in White *et al*.^19^ To allow for *operando* imaging, a support base was designed and printed by fusion deposition modelling using a Stratasys uPrint SE with ABSPlus material. This support base allowed for secure attachment of the small-scale fixture to the sample stage within a Zeiss Xradia Versa 520 XCT system, as well as provide necessary compression for proper fuel cell operation. The support base also allowed for electrical connections and exhaust gas line management from the fixture. To stop impingement of exhaust gas tubing and electrical connections during rotation of the sample base while imaging, a cable management system was used which was installed in the XCT enclosure by Zeiss. Inlet gas lines, which can be seen in Fig. 2, were supported from the ceiling of the XCT enclosure so as not to interrupt the X-ray beam path during imaging. Because of this, the tubing needed to be flexible and long enough to allow for smooth rotation; for this, high-temperature soft Viton fluoroelastomer was used. Insulated and temperature-controlled gas lines were used to connect the fixture inside the XCT system to the fuel cell test station which was situated externally to the XCT system. The heated gas lines were managed using a custom aluminum framing designed to avoid intervention of XCT system function. Additionally, a hydrogen sensor and a smoke detector were installed on this framing to allow for safe operation and shutdown in the event of a significant leak or ignition event during *operando* imaging. Inlet/outlet gas lines and electrical connections were fed outside the XCT enclosure through a specially designed side baffle with two orthogonal turns in order to prevent stray X-ray radiation by eliminating a direct line-of-sight exit to the lab environment. This opening and the additional attached safety cover on the Versa 520 system was supplied by Zeiss.

The control of the fuel cell operation, gas humidity and electrical measurement was affected by a Scribner 850C Fuel Cell Test System. Details concerning the fuel cell conditioning, diagnostic measurements and AST operation and conditions have been reported elsewhere in White *et al*.^19^. The AST was performed by square wave voltage cycling of 1.4 V upper potential (60 s hold) and 0.6 V (30 s hold) lower potential. The MEA was fabricated using the catalyst coated membrane method, with 50:50 Pt/C ratio at 0.4/0.1 mg cm^−2^ Pt loadings and 23 weight percent ionomer for cathode and anode electrodes, respectively. The catalyst layers were coated onto a Dupont Nafion® NR211 membrane with GDLs consisting of teflonated Avcarb® non-woven carbon paper coated with a micro-porous layer (MPL). The voltage cycling was performed outside the XCT system to allow for accurate temperature and humidity control by minimal gas line length and insulation as well as to free the XCT system for other imaging tasks; during *operando* imaging, the fuel cell was operated at room temperature conditions inside the XCT enclosure. This low temperature operation limited the amount of humidity that the input gases into the fuel cell carried and the liquid water observed directly results from the fuel cell electrochemical reaction, allowing for improved imaging of transient water distributions without contribution from inlet gas humidity or condensation along the gas lines.

The operational workflow can be itemized as follows: Conditioning and beginning-of-life (BOL) diagnostic measurements, such as polarization curves and EPSA measurement, were taken outside the XCT system with the small-scale fixture directly attached to the test station; small-scale fixture purged with ‘dry’ (100% RH at 25 °C) N_2_ to remove all excess water and residual reactant gases; fixture moved into the XCT system and re-connected to gas lines and electrical connections leading to the test station; small-scale fixture and gas lines purged with N_2_ for additional 30 min; H_2_/air supplied to small-scale fixture and allowed to equilibrate while drawing a current density of 250 mA/cm^2^ at room temperature conditions; after 30 min equilibration, imaging performed while fuel cell in operation with details of imaging parameters outlined in White *et al*.^19^; subsequent imaging at current densities of 500 mA/cm^2^ and 750 mA/cm^2^ allowing for 30 min equilibration at each current density before imaging; following imaging, fixture purged with N_2_ to remove all excess water for a minimum of 4 h; ‘dry’ image set collected with continuous N_2_ flow; fixture taken out of the XCT system and subjected to an AST protocol; and diagnostic measurements subsequently performed after cycling with the previously outlined imaging steps repeated. This workflow extended the same location tracking of cathode catalyst degradation with GDL water distribution visualization at various current densities which has not been previously reported. The total imaging time was minimized to reduce the impact of X-ray irradiation on the fuel cell materials, with additional low energy X-ray filter used (proprietary and provided by Zeiss), and falls within the expected non-destructive impact time determined previously by White *et al*.^45^.

### Post-processing

#### GDL water segmentation

Following image acquisition, the set of radiographs was reconstructed using Zeiss proprietary software, which computes the 3D representation of the imaged area. To properly compare greyscale values of the resulting image stacks across multiple acquisition stages, the byte scaling was set consistently to the same value across all reconstructions. This results in the same pixel value for constant materials such as air and graphite plate material, which were unchanged throughout the experiment. To accurately segment liquid water from the pores of the GDL, it was necessary to subtract the ‘wet’ image set (obtained during current collection) from the ‘dry’ reference (no current, N_2_ flow); however, prior to this step, it was crucial to properly align image sets in x, y and z directions. First, the image stacks were cropped to the region of interest to reduce unnecessary processing time and minimize data storage. Second, all image stacks were aligned to the beginning-of-life ‘dry’ image set using custom macros written in ImageJ macro language^46^. The macros allow the user to correct for in-plane tilt and align in x, y and z by comparing the standard deviation of the resultant subtracted image; the image pair with the smallest standard deviation is chosen to represent the translation and slice number of the aligned image stacks, which can then be applied manually after inspection. Following subtraction of the two image sets, 3D median filter as well as 3D anisotropic filter is used^47–49^. The result is then processed in Matlab using Otsu threshold to create binary images of the resultant water droplets. Noise and GDL fibers are also removed using a custom processing script comparing eccentricity and size of the labelled objects^34^. These steps were repeated for all image stacks using the same parameters to keep consistency when comparing values across multiple image acquisition stages.

#### Cathode catalyst layer and membrane segmentation

A custom segmentation procedure, written in ImageJ macro language is used to obtain an accurate representation of the CCL and quantitatively determine morphological features such as thickness, crack area, and crack width. The script allows for automated separation of the cathode layer following thresholding by the user by Gaussian Mixed Model^19^, as well as 3D particle labelling using BoneJ plugin^50^. An overlay of the result from the segmentation and the accuracy is shown in Supplementary Information (SF1). The cathode separation script can also be found on the project repository on FCReL website. Membrane segmentation is obtained following separation of the anode and CCLs. Once both layers are segmented by the user using the aforementioned script, additional scripts written in Python (programming language, www.python.org) using NumPy (scientific computing package, www.numpy.org) as well as SciPy (scientific computing package, www.scipy.org) separate the membrane by using the boundary of the catalyst layers and nearest-neighbor interpolation to be robust against cracks or breaks in the segmented catalyst layers. This method of segmentation is required since the greyscale values of the membrane layer are similar to those of the carbon in the GDL, and noise levels make using texture filters such as entropy ineffective.

#### Composition calculation from cathode catalyst layer greyscale values

The X-ray attenuation of catalyst layer material changes following degradation, which can be observed directly by changes in pixel greyscale values^19,51^. To quantify these changes, a greyscale calculation model was implemented. This model takes as input the greyscale values from a 2D image of the CCL and determines the corresponding material composition. To perform such a calculation, the following steps are required. First, imaging parameters and catalyst layer size are set such that minimal local tomography is performed, as this can affect greyscale values of the reconstruction output^52^. Second, the laboratory scale XCT system produces a non-monochromatic X-ray beam for imaging; thus, filters are used to narrow the energy distribution such that a single ‘effective’ energy is used in the calculation. This is an approximation, and the authors note that improved results could be obtained with the use of a monochromatic beam. Lastly, a set of materials with known density and composition is used to calibrate and correlate greyscale value with X-ray linear attenuation values. The calibration curve obtained is shown in Supplementary Information (SF4) and follows an expected linear trend, indicating that a single energy assumption is valid in this case. With these experimental considerations, a model can be implemented. The calculation required is as follows:

$$GSV={m}_{{\rm{cal}}}\cdot {\mu }_{{C}_{x}{H}_{x}{F}_{x}{O}_{x}{S}_{x}P{t}_{x}}+{b}_{{\rm{cal}}}$$

where m_cal_ and b_cal_ are slope and intercept from the calibration curve, respectively, and ${\mu }_{{C}_{x}{H}_{x}{F}_{x}{O}_{x}{S}_{x}P{t}_{x}}$ is the linear attenuation coefficient from the constituents of the catalyst layer. NIST provides an online calculation to determine the mass attenuation of a composition of materials at a specified energy (https://physics.nist.gov/PhysRefData/FFast/html/form.html). However, for each new composition, a user must manually retrieve the corresponding mass attenuation value. Since the catalyst layer composition is unknown and varies widely, this is however not practical. Instead, a collection of one hundred values with uniform variation in fractional composition of carbon, platinum and ionomer (H, F, O, S) was collected. From this dataset, an implicit model was fitted using Support Vector Machine (SVM) regression with radial basis function from scikit-learn (http://scikit-learn.org), an open-source, python based library for artificial intelligence. To obtain linear attenuation values, the mass attenuation determined from the implicit model was multiplied by the density, which was calculated using the local thickness determined after segmentation of the CCL. Using this model, a constrained optimization routine was then implemented to minimize the difference between the experimental greyscale pixel value and the calculated value, as detailed in the Supplementary information (SF5). The optimization routine uses constrained optimization by linear approximation (COBYLA) from scipy library. The constraint function is the porosity calculation limited between 0 and 1:

$$\varphi =1-\frac{{\rho }_{bulk}}{{\rho }_{particle}}$$

where void space is assumed to be filled with air; *ρ*_*bulk*_ is the calculated density from composition obtained from the optimization routine and local thickness; and *ρ*_*particle*_ is calculated based on the known densities for carbon (graphite), platinum and ionomer, as listed in Table 3. The experimental greyscale values were obtained from the zero-current density (‘dry’) image set and projecting average through-plane greyscale values to a single 2D image after segmentation of the CCL. For beginning-of-life (0 AST cycles), nominal values of the CCL composition were given as initial guess for the optimization routine. These are listed in Table 3 as *Nominal*_*x*_. The ionomer content was assumed to not change following the presently used AST cycling, and was not altered during the subsequent optimization routine from 0 AST cycles, while carbon and platinum were free to change due to degradation. Initial values were taken from the 0 AST cycles model output such that the local variation in ionomer, Pt and C was considered. That is, the model was applied to the BOL catalyst layer to determine local variation in ionomer, C and Pt content, while calculations following AST cycles used these local values as inputs and adjusted the C and Pt content to match the greyscale values obtained from the experiment.

**Table 3:** Input parameters used for quantification of CCL composition changes from the datasets of pixel greyscale values obtained through XCT.

Greyscale model input parameter	Value
X-ray energy	32.5 keV
*ρ*_*c*_	2.3 g cm^−3^
*ρ*_*pt*_	21.5 g cm^−3^
*ρ*_*Ionomer*_^53^	2.0 g cm^−3^
*Nominal*_*Pt*_	0.4 mg cm^−2^
*Nominal*_*C*_	50 wt%
*Nominal*_Ionomer_	23 wt%

## Supplementary information

Supplementary Information
