# Supplementary material for: Four-dimensional joint visualization of electrode degradation and liquid water distribution inside operating polymer electrolyte fuel cells
Source: Sci Rep. 2019 Feb 12;9:1843. doi: 10.1038/s41598-018-38464-9 (PMC6372714; doi:10.1038/s41598-018-38464-9)
Supplement: Supplementary file 1 — Supplementary Information [file 41598_2018_38464_MOESM1_ESM.pdf]

Four-dimensional joint visualization of electrode degradation and liquid water distribution inside operating polymer electrolyte fuel cells

Robin T. White[1], Sebastian H. Eberhardt[1], Yadvinder Singh[1], Tylynn Haddow[1], Monica Dutta[2], Francesco P. Orfino[1], Erik Kjeang[1,*]

[1]Fuel Cell Research Laboratory (FCReL), School of Mechatronic Systems Engineering, Simon Fraser University, 250-13450 102 Avenue, Surrey, BC, V3T 0A3, Canada
[2]Ballard Power Systems, 9000 Glenlyon Parkway, Burnaby, BC, V5J 5J8, Canada
*ekjeang@sfu.ca

Supplementary data

Porosity is determined by segmentation of the cathode GDL structure from 'dry' beginning-of-life and is shown for the specific areas under land and channel in Figure S1. A noticeable decrease in porosity is observed for the region of approximately 15 μm below the edge of the land, indicating compression reduced pore size. Higher variation under the land also indicates variation in pore sizes dependent on the reaction of GDL regions under compression; that is, how much the compression pressure affects that particular region of the GDL. Additionally, the impact of compression is partially extended to the channel region, as seen by the small dip in porosity after the start of the land (dotted line in Figure S1). The GDL region close to the catalyst layer including certain portions of the microporous layer (MPL) cannot be accurately segmented due to the limit on resolution and is hence treated as solid.

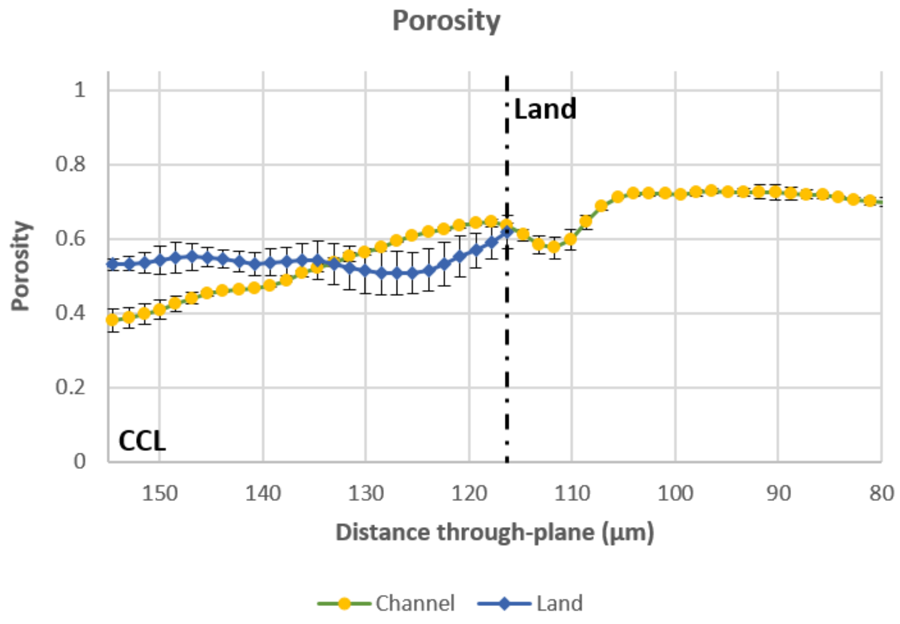

Figure S1. Porosity measurement of the cathode gas diffusion layer under land and channel regions from segmentation of the XCT image dataset obtained in the 'dry' beginning-of-life state.

As shown in Figure S2 for a fixed current density, the liquid water distribution in the cathode GDL was generally consistent across degradation stages, featuring similar droplets and areas of flooding. This indicates good reproducibility in regards to the experimental technique while also highlighting the stark absence of liquid water in the cathode GDL after 750 cycles due to severe degradation.

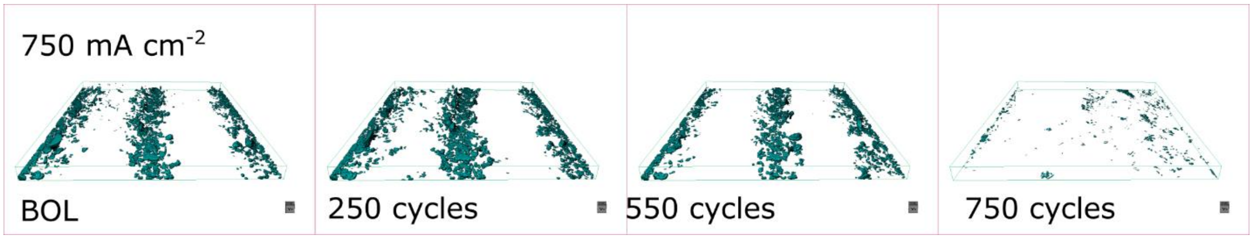

Figure S2. Cathode GDL liquid water distribution visualization at 750 mA cm^{-2} current density at different stages of degradation. Consistency in droplet locations indicates preferred water transport pathways and areas prone to flooding. However, significantly less water is observed after 750 cycles.

Supplementary methods

Cathode catalyst layer and membrane segmentation

Cathode catalyst layer segmentation and accurate thickness measurement is important to properly quantify changes resulting from carbon corrosion. Shown below in Figure S2 are outlines of segmentation regions and thickness measurements indicating the accuracy at 1.5 µm pixel size resolution used for *operando* imaging. As can be seen, from the custom macros written in ImageJ, distinct separation of the cathode catalyst layer from all other MEA components allows for accurate

thickness measurements in good agreement with higher resolution XCT imaging (0.69 µm pixel size) and SEM imaging data.

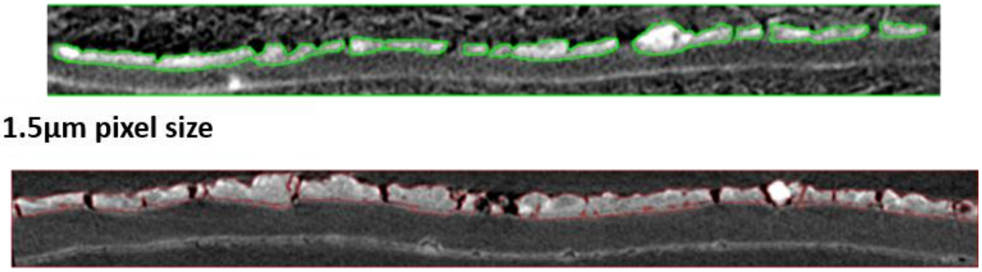

0.69µm pixel size

Average thickness Green: 11.6 ± 3.8µm (9mm^2 area)

Average thickness Red: 11.0 ± 3.9µm (0.42mm^2 area)

SEM thickness: 11.8 ± 3.3µm

Figure S3. Cross-sectional outlines of the cathode catalyst layer segmentation used to determine local thickness quantification for two different MEAs under different XCT resolution measurements showing good agreement with SEM data, hence validating the quantification procedure.

Composition calculation from cathode catalyst layer greyscale values

A calibration curve to determine the conversion of linear attenuation to greyscale value was obtained by imaging known materials and fitting to a linear curve where the energy used allows a straight line to join all points within experimental uncertainty. Shown below in Figure S3 is the calibration curve for four known materials (shown in blue) and nominal cathode catalyst layer value (shown in orange) using an effective X-ray energy of 32.5 keV. This is the mean energy after applying filters to the X-ray beam to remove the low energy contributions. The known materials were imaged using a custom calibration puck which was composed of the same material as the small-scale fixture as well as the same dimensions and imaging parameters. The materials used were air, flow field graphite plate, sulfuric acid and titanium foil. These materials gave a good distribution of values to properly determine an accurate calibration curve when used in the greyscale model. Variation in the cathode catalyst layer greyscale value can then be converted to linear attenuation, and with known thickness from segmentation, density and composition can then be inferred.

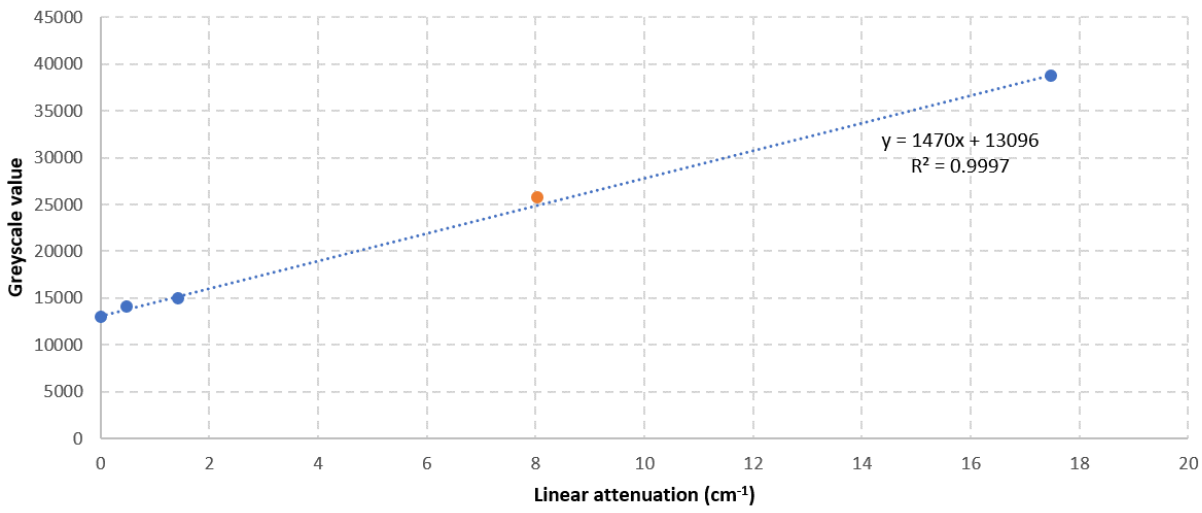

Figure S4. Calibration curve for greyscale value and linear attenuation of materials to be used as conversion in the model calculation. The known materials used in the calibration are air, flow field graphite plate, sulfuric acid and titanium foil (blue, lowest to highest attenuation values). The cathode catalyst layer with nominal composition and thickness is shown in orange.

To calculate the mass attenuation of a composition of the cathode catalyst layer, values obtained from NIST (https://physics.nist.gov/PhysRefData/FFast/html/form.html) were fitted to an implicit model. The obtained values from NIST are shown in Figure S4a as data points with the implicit model shown as the color area fit. The fitted model has an accuracy of 0.15 cm^2 g^{-1} which is less than 1.5% error for nominal cathode catalyst layer composition values. From the experimentally obtained greyscale values by XCT imaging, linear attenuation can be determined from the above calibration curve. A constrained optimization routine was run to determine the composition of the cathode catalyst layer that will give rise to this greyscale, where the proposed composition was obtained when the difference between calculated greyscale and experimentally obtained greyscale was minimized. This is shown in Figure S4b with the constraint boundary in black and the obtained optimization point shown at a mass attenuation value of 7.79 cm^2 g^{-1} and density of 0.97 g cm^{-3}, which falls close to the initial guess (nominal) values for composition. Due to the fact that higher ionomer content will increase the density by filling pores as well as reduce the mass attenuation value, there is not one single value for composition that can yield the same greyscale. This is a limitation of this calculation and comparison with energy dispersive spectroscopy (EDS) has been used for validation. Typically, high ionomer regions have been observed to be very thick from dense agglomerates forming and have been used as a que for possible high ionomer regions during the calculation. The model will typically find composition that is close to the nominal values used for initial guess and thus cannot be used for an unknown material; however, as shown in this report, it can be used to determine variation around this composition and changes resulting from corrosion.

a

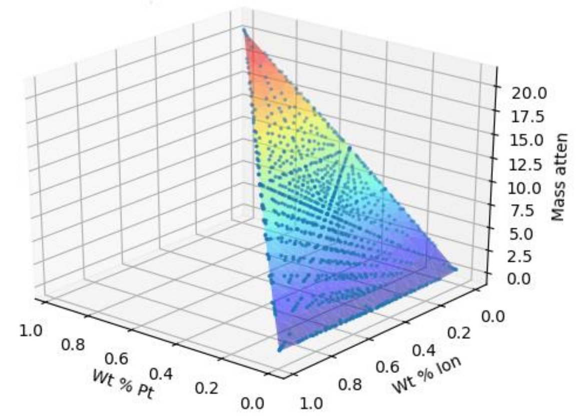

b

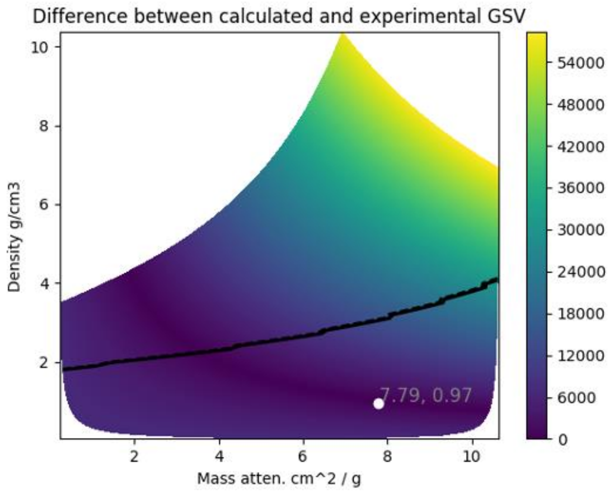

Figure S5. (a) Implicit model and obtained mass attenuation values from NIST for variation in Pt, carbon (not shown) and ionomer composition to be used in greyscale model calculation. (b) Plot of the optimization search space with the constraint function shown in black. Values below this line are possible composition values, with the found optimization point indicated by the white dot which falls close to initial nominal values.
